# Evidence that the novel receptor FGFRL1 signals indirectly via FGFR1

**DOI:** 10.3892/ijmm.2013.1484

**Published:** 2013-09-10

**Authors:** RUTH AMANN, BEAT TRUEB

**Affiliations:** 1 Department of Clinical Research, University of Bern, 3010 Bern, Switzerland; 2 Department of Rheumatology, University Hospital Bern, 3010 Bern, Switzerland

**Keywords:** fibroblast growth factor, fibroblast growth factor receptor, fibroblast growth factor receptor-like protein 1, kidney development, renal vesicle, nephron

## Abstract

Fibroblast growth factor (FGF) receptor-like protein 1 (FGFRL1) is a recently discovered member of the FGF receptor (FGFR) family. Similar to the classical FGFRs, it contains three extracellular immunoglobulin-like domains and interacts with FGF ligands. However, in contrast to the classical receptors, it does not contain any intracellular tyrosine kinase domain and consequently cannot signal by transphosphorylation. In mouse kidneys, FgfrL1 is expressed primarily at embryonic stages E14–E15 in regions where nascent nephrons develop. In this study, we used whole-mount *in situ* hybridization to show the spatial pattern of five different Fgfrs in the developing mouse kidney. We compared the expression pattern of FgfrL1 with that of other Fgfrs. The expression pattern of FgfrL1 closely resembled that of Fgfr1, but clearly differed from that of Fgfr2–Fgfr4. It is therefore conceivable that FgfrL1 signals indirectly via Fgfr1. The mechanisms by which FgfrL1 affects the activity of Fgfr1 remain to be elucidated.

## Introduction

The fibroblast growth factor signaling system plays an important role in the development of most multicellular animals. It controls the proliferation, differentiation, migration and apoptosis of virtually all cell types. The genomes of mice and humans code for 22 different FGF ligands (FGF1–FGF23, FGF15=FGF19) that can interact with four different FGF receptors (FGFRs; FGFR1–FGFR4) (1,2). The FGFs bind together with heparan sulfate to either one of the four receptors and trigger, via transphosphorylation, several intracellular signaling cascades, such as the mitogen-activated protein kinase (MAPK)/Erk, the phosphoinositide 3-kinase (PI3K)/Akt, the Jak/Stat and the phosphoinositide phospholipase C (PLC)γ pathway.

All FGFRs contain three extracellular immunoglobulin-like domains (Ig domains 1–3), a single transmembrane domain and an intracellular tyrosine kinase domain (2). Alternative splicing contributes to the complexity of the system. Each of the receptors FGFR1, FGFR2 and FGFR3 occurs in two different splice variants that differ by the precise amino acid sequence of Ig domain 3. At the genomic level, this domain is encoded by three different exons, namely exons IIIa, IIIb and IIIc. Exon IIIa codes for the first half of Ig domain 3 and is used for all splice variants, but exons IIIb and IIIc are used in a mutually exclusive manner to give rise to two different splice variants, the b and the c variants. A total of seven different receptors can therefore be generated, FGFR1b, FGFR1c, FGFR2b, FGFR2c, FGFR3b, FGFR3c and FGFR4 (1,2). The b splice variants are primarily expressed in epithelial tissues, the c variants mainly in mesenchymal tissues.

The metanephric (permanent) kidney of mammals is formed by two different tissues, the metanephric mesenchyme and the ureteric bud (3,4). In mice, the development of the metanephric kidney begins on embryonic day E10.5 when the ureteric bud invades the metanephric mesenchyme. By a series of reciprocal interactions, the metanephric mesenchyme induces the ureteric bud to branch in a stereotypical fashion, while the ureteric bud induces the metanephric mesenchyme to condense around its tips and to undergo a mesenchymal-to-epithelial conversion that leads to the formation of renal vesicles. The renal vesicles develop further into comma- and s-shaped bodies and finally form functional nephrons. Gene expression profiling has revealed that the developing mouse kidney expresses Fgfs 1, 7, 8, 9, 10, 12 and 20 (5).

In the year 2000, we discovered a fifth FGFR that we termed FGF receptor-like protein 1 (FGFRL1) (6). This receptor contains three Ig domains and a single transmembrane domain similar to the classical FGFRs. However, it lacks the tyrosine kinase domain and instead contains a short unrelated sequence at the intracellular side that ends with a histidine-rich domain (7–10). FGFRL1 is expressed primarily in cartilage and developing bones, and at lower levels in many other organs including kidneys and muscles. Mice with a targeted disruption of the novel receptor-like gene (knockout mice) develop to term and are born alive (11,12). However, these animals die immediately after birth and show severe kidney dysgenesis or kidney agenesis due to the lack of renal vesicles (13). We confirmed that FgfrL1 is in fact expressed in renal vesicles and all nephrogenic structures during the early steps of kidney development (14,15).

The molecular mechanisms behind the involvement of FGFRL1 in FGF signaling have not yet been elucidated. It cannot signal by transphosphorylation as it does not contain any intracellular tyrosine kinase domain. When mRNA for FGFRL1 was injected into blastomers of *Xenopus* embryos, it interfered with FGF signaling and led to gastrulation defects that affected the trunk and tail of the embryos (16). This effect was overcome by the co-injection of mRNA for FGFR1. We therefore concluded that FGFRL1 may act as a decoy receptor that binds and neutralizes FGF ligands. However, this hypothesis was challenged by more recent findings obtained with FgfrL1 null mice. A comparison of the mRNA profiles from wild-type and knockout animals using gene microarrays revealed that the lack of FgfrL1 was not compensated for by another Fgfr or by any downstream signaling molecule (14). Furthermore, the phenotype of our knockout animals was strikingly similar to the phenotype of animals with a conditional deletion of Fgf8, which also lack any nephrons in their metanephric kidneys (13,17,18). If FgfrL1 served as a simple decoy receptor for Fgf ligands one would expect to observe more, and not less Fgf signaling in our knockout animals and consequently an increased, rather than a decreased, number of nephrogenic structures and/or ureteric buds.

To gain a better understanding of the working mechanisms of FgfrL1, we decided to compare the exact expression pattern of FgfrL1 with that of the other Fgfrs. We hypothesized that a particular receptor would show an expression pattern similar to that of FgfrL1 if FgfrL1 is involved, directly or indirectly, in its signaling cascade. We found that the FgfrL1 expression pattern greatly resembled that of Fgfr1, but clearly differed from that of Fgfr2–Fgfr4, suggesting that FgfrL1 may participate in Fgfr1 signaling.

## Materials and methods

### Animals

Kidneys were obtained from mice (strain C57BL/6) bred at our local animal facility. For a timed pregnancy, the noon of the day, at which a vaginal plug could be detected, was counted as E0.5. All animal experiments were approved by the Swiss Federal Veterinary Office (BVET) (BE84/12).

### RNA preparation and northern blotting

Kidneys were dissected from mouse embryos and immediately placed into RNA-later buffer (Sigma, St. Louis, MO, USA). In order to obtain enough RNA, kidney rudiments of early developmental stages (E12.5–E14.5) were pooled from 20–30 individual embryos. RNA was prepared using the GeneElute mammalian total RNA kit from Sigma and separated on 1% agarose gels in the presence of 1 M formaldehyde. The resolved bands were transferred from the gel to a nylon membrane by vacuum blotting. The membrane was hybridized at 42°C with radioactively labeled cDNA probes in a buffer containing 50% formamide. These probes were labeled by the random primed oligolabeling method with [α-^32^P] dCTP. After overnight hybridization, the blot was washed at regular stringency [1X standard saline citrate (SSC)] and exposed to X-ray film (Carestream Kodak BioMax MS; Sigma).

Hybridization probes for the canonical Fgfrs were generated by PCR utilizing cDNA prepared from E16.5 mouse kidneys and the primer pairs listed in Table I. Probes for Fgfr1–Fgfr3 were selected in a manner that they hybridized equally well with the b and the c splice variants. The PCR fragments were inserted into the *Bam*HI/*Xba*I site of the expression vector pSPT19 (Roche Applied Science, Basel, Switzerland). The probe for FgfrL1 corresponded to an *Xba*I/*Bam*HI fragment derived from the full-length cDNA sequence (9), which was subcloned into pSPT19. The final probes encompassed nucleotides 766–1554 (corresponding to amino acids 7–269) of Fgfr1 (NM_010206), nucleotides 1336–1979 (amino acids 57–271) of Fgfr2 (NM_010207), nucleotides 374–1093 (amino acids 23–262) of Fgfr3 (NM_008010), nucleotides 503–1242 (amino acids 115–360) of Fgfr4 (NM_008011), nucleotides 661–1417 (amino acids 190–441) of FgfrL1 (AJ293947) and nucleotides 211–899 (amino acids 26–255) of calbindin (NM_009788). The reading frame and authenticity of all constructs were verified by DNA sequencing.

### Whole-mount in situ hybridization

Whole-mount in situ hybridization was performed using E15.5 mouse kidneys following the protocol provided in the GUDMAP gene expression database (http://www.gudmap.org/Research/Protocols/McMahon.html). Riboprobes were generated from cDNA sequences cloned into pSPT19 (see above) by transcription in the presence of digoxigenin-labeled UTP using the SP6/T7 DIG RNA Labeling kit from Roche as previously described (13). Kidneys were dissected from 15.5-day-old embryos and fixed overnight with 4% paraformaldehyde (PFA). The tissue was dehydrated by serial treatment with methanol/phosphate buffer (25, 50, 75 and 100% methanol in PBS containing 0.1% Tween-20). The specimens were rehydrated, bleached for 30 min with 6% hydrogen peroxide and digested for 15 min with 10 μg/ml proteinase K. Subsequently, the samples were fixed with 0.2% glutaraldehyde/4% PFA, pre-hybridized at 68°C for 2 h in hybridization buffer (50% formamide, 5X SSC, 1% SDS, 50 μg/ml t-RNA from yeast, 50 μg/ml heparin) and then hybridized overnight with the riboprobes. Following washing with 50% formamide, 5X SSC, 1% SDS at 65°C, the samples were blocked for 2 h at room temperature with 3% bovine serum albumin in Tris-buffered saline and then incubated with anti-digoxigenin antibodies conjugated with alkaline phosphatase (Roche, 1:2,000). After extensive washing, the hybridization signal was developed with BM Purple (Roche) for 7–78 h.

## Results

### Analysis of Fgfr expression by northern blotting

The expression of the four classical receptors was analyzed by northern blotting with samples from embryonic kidneys at stage E15.5. Special care was taken so that the four hybridization probes had a similar specific radioactivity to allow the direct comparison of the resulting hybridization signals between the four probes. Moreover, the sequences of the probes were selected in a manner that the probes hybridized equally well with the b and the c splice variants of the receptors.

**Figure 1:**
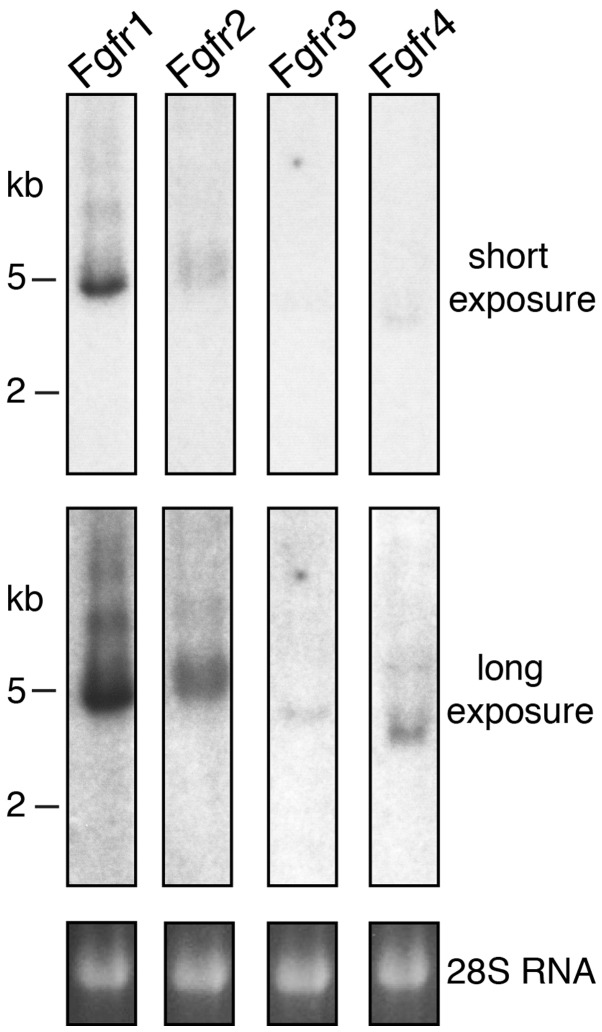
Analysis of fibroblast growth factor receptor (Fgfr) expression in the mouse kidney. Aliquots (10 μg) of total RNA from mouse kidneys at E15.5 were resolved in parallel on an agarose gel, transferred to a nylon membrane and individually hybridized with radiolabeled probes for Fgfr1, Fgfr2, Fgfr3 and Fgfr4 as indicated. The migration positions of the ribosomal RNAs are indicated in the margin. As a loading control, the ethidium bromide stained 28S ribosomal RNA is shown.

A particularly strong hybridization signal was obtained with the probe for Fgfr1 (Fig. 1). This result suggests that Fgfr1 is the principal receptor in the developing mouse kidney at stage E15.5. A strong signal was also observed with the probe for Fgfr2. However, in this case the resulting band was broader and fuzzier, possibly due to the presence of several distinct mRNA splice variants and/or different polyadenylation sites. By contrast, the signals for Fgfr3 and Fgfr4 were extremely weak and could be detected only after extended exposure of the northern blots, indicating that these receptors are expressed in the kidneys at very low levels during early development. The electrophoretic mobility of all the positive bands was consistent with the size of the four mRNAs predicted from their cDNA sequences (Fgfr1 5,000 bp, Fgfr2 5,200 bp, Fgfr3 4,500 bp and Fgfr4 3,500 bp). Our northern blotting experiment also demonstrated that no unexpected crossreaction occurred between each of the probes and the four receptors.

**Figure 2:**
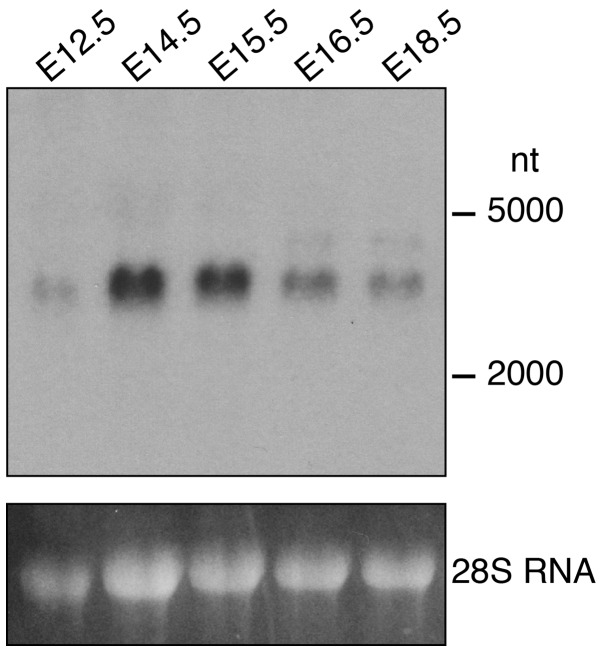
Analysis of FgfrL1 expression during early kidney development. Aliquots of total RNA from mouse kidneys of different developmental stages were resolved on an agarose gel, transferred to a nylon membrane and hybridized with a probe for FgfrL1. The migration positions of the ribosomal RNAs are indicated in the margin. As a loading control, the ethidium bromide stained 28S ribosomal RNA is shown.

The expression of FgfrL1 was analyzed on a separate northern blot containing RNA from kidneys at five different developmental stages (Fig. 2). A band of 3,000 nt was observed consistent with the published size of the mouse FgfrL1 mRNA (8,9). This band was barely detectable at stage E12.5 but became clearly visible at E14.5 and E15.5 and decreased thereafter. This result suggests that FgfrL1 is required during the early stages of kidney development when renal vesicles and comma- and s-shaped bodies develop (3,4).

### Analysis of Fgfr expression by in situ hybridization

**Figure 3:**
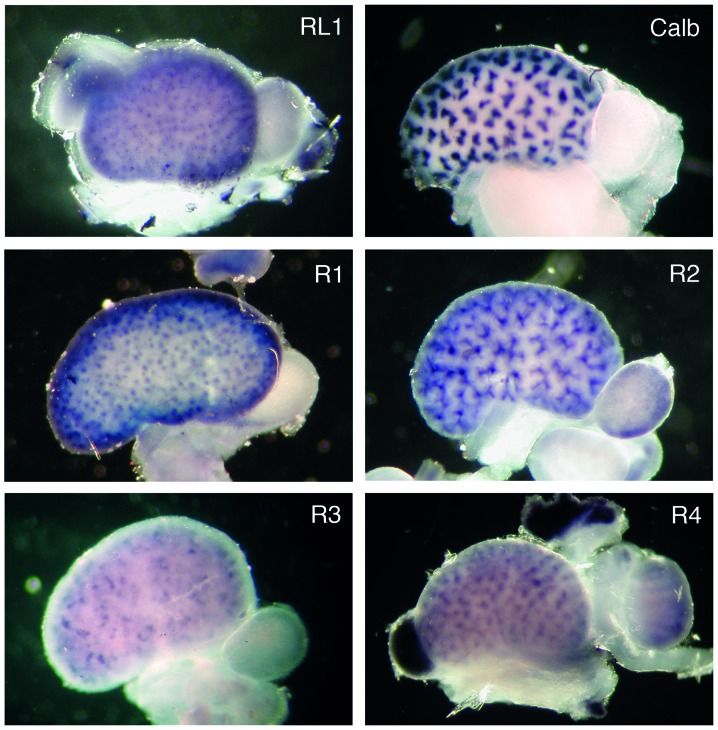
Expression patterns of the five fibroblast growth factor receptors (Fgfrs) in mouse kidneys. Kidneys at stage E15.5 were stained by whole-mount *in situ* hybridization with riboprobes for Fgfr1 (R1), Fgfr2 (R2), Fgfr3 (R3), Fgfr4 (R4), FgfrL1 (RL1) and calbindin 1 (Calb) as indicated. The results demonstrated that the expression pattern of FgfrL1 looked very similar to that of Fgfr1 and the pattern of Fgfr2 similar to that of calbindin. Fgfr3 and Fgfr4 were expressed at very low levels and revealed more complex expression patterns.

The spacial distribution of the FgfrL1 mRNA was compared with the distribution of the four classical receptors by whole-mount *in situ* hybridization of entire kidneys at stage E15.5 (Fig. 3). The cDNA sequences that had been used above for the northern blotting experiment were utilized for the preparation of biotinylated riboprobes. The hybridization of a control kidney with a probe for calbindin yielded a pattern of clover leaf-like structures consistent with the expression of this marker gene in all ureteric buds. Hybridization of a kidney with a probe for FgfrL1 produced a completely different pattern. A fine punctate or dotted distribution was observed, which was consistent with expression of the FgfrL1 gene in renal vesicles and nascent nephrogenic structures. Hybridization with a probe for Fgfr1 produced a very similar pattern, suggesting that the expression of Fgfr1 and FgfrL1 overlapped to a large extent. Hybridization with a probe for Fgfr2 revealed a pattern resembling the distribution of calbindin. However, the signal of Fgfr2 was more diffuse, particularly at the ureteric tips, suggesting that Fgfr2 expression was not confined exclusively to the ureteric bud, but occurred to some extent also in nascent nephrons. The signals obtained with the probes for Fgfr3 and Fgfr4 were extremely weak. They both revealed distributions that were more complex than the above-mentioned expression patterns.

The progression of the enzymatic reaction that was used for development of the signal shown in Fig. 3 may provide a crude measure of the relative expression levels of the four receptors. A robust signal was obtained after 7 h with the probe for Fgfr1 and after 20 h with that for Fgfr2, indicating that these two receptors are the major Fgf signaling proteins in the developing kidney at E15.5. To obtain a signal for Fgfr3 and Fgfr4, the color reaction had to proceed for 78 h, again suggesting that these receptors are expressed at extremely low levels. The development time for FgfrL1 was 19 h, comparable to that of Fgfr2.

## Discussion

In this study, we used the whole-mount *in situ* hybridization technique to show the spacial expression pattern of five different Fgfrs in the developing mouse kidney. This technique yields information about the three-dimensional distribution of the corresponding mRNAs in the cortex of the kidney, whereas *in situ* hybridization of thin sections would show only a two-dimensional expression pattern. Whole-mount *in situ* hybridization appears to be more sensitive than section hybridization as it accumulates signals from the depth of the kidney cortex, which offers an extra advantage when expression levels are very low. In fact, section *in situ* hybridization did not yield convincing data with probes for Fgfr3 and Fgfr4. Moreover, the signal obtained with FgfrL1, the fifth Fgfr, proved to be extremely weak by section hybridization and had to be electronically enhanced for visualization in a previous publication (14). By contrast, whole-mount *in situ* hybridization with FgfrL1 yielded robust signals that did not have to be enhanced.

In this study, we demonstrate that Fgfr1 and Fgfr2 are the major receptors of the Fgf signaling system expressed in the early stages of developing kidneys. On the other hand, the expression of Fgfr3 and Fgfr4 was very low, raising doubts about the functional significance of these receptors during kidney development. This observation is in accordance with the results obtained from experiments using knockout mice (Table II). The disruption of the genes for Fgfr3 and Fgfr4, either alone or in concert, did not produce any altered phenotype in the mouse kidneys (19,20). Fgfr3 and Fgfr4 knockout animals were viable and only Fgfr3-null mice showed an obvious phenotype with abnormally long bones (19). By contrast, the disruption of each of the receptors Fgfr1 and Fgfr2 caused lethality at very early embryonic stages before nephrogenesis was initiated, preventing the analysis of these genes during kidney formation (21–24). Thus, a conditional targeting approach had to be used for these cases. Interestingly, the conditional disruption of Fgfr1 in the metanephric mesenchyme or in the ureteric bud did not yield any overt phenotype (25–27). Likewise, the conditional deletion of Fgfr2 in the metanephric mesenchyme did not produce any severe alterations (25). Only the conditional deletion of Fgfr2 in the ureteric bud produced animals with abnormalities in ureteric branching, but the phenotype was relatively mild and the animals were viable (26,27). The phenotype was more severe when both receptors were deleted in concert. After the compound deletion of Fgfr1 and Fgfr2, no metanephric mesenchyme formed, suggesting that either Fgfr1 or Fgfr2 is required for nephrogenesis but that the two receptors can substitute for one another (25,27). The observations made with the four classical receptors are in sharp contrast with the targeted disruption of the fifth receptor. The global deletion of FgfrL1 yielded animals that specifically lacked the metanephric kidneys and died at birth (13). At E10.5, the ureteric bud of these animals still invaded the metanephric mesenchyme, but branching stopped after the T-state and no renal vesicles were formed. It is intriguing to note that the targeted disruption of FgfrL1, but not of any other Fgfr, completely inhibited kidney development, although FgfrL1 is expressed at very low levels in the kidneys.

In this study, we demonstrated that the spacial distribution of FgfrL1 mRNA closely resembled that of Fgfr1, but clearly differed from that of Fgfr2–Fgfr4. FgfrL1 cannot signal on its own as it lacks the intracellular tyrosine kinase domain. Since it still interacts with Fgf ligands, it is likely that it indirectly modulates the Fgf signaling of another receptor. Thus, we concluded that this other receptor may be Fgfr1, since only this receptor shows a similar distribution in developing kidneys.

Originally we (7,16), as well as others (8) have speculated that FgfrL1 may act as a decoy receptor that binds and neutralizes Fgf ligands. However, recent results obtained by DNA microarray profiling suggest the opposite (14). The disruption of the FgfrL1 gene in mice was not accompanied by the specific upregulation of any target genes that are known to be controlled by Fgf signaling. Yet, such an upregulation would be expected if FgfrL1 acted as a simple decoy receptor. By contrast, we found that approximately 50 gene products were significantly downregulated upon the disruption of FgfrL1 expression, including wingless-type MMTV integration site family member 4 (Wnt4), dickkopf 1 (Dkk1), early growth response 1 (Egr1), Fgf8 and LIM homeobox 1 (Lhx1) (14). It is therefore likely that FgfrL1 acts as a positive regulator of Fgf signaling, rather than as a decoy receptor, at least in the kidneys.

It has been demonstrated that the developing kidneys of mice express Fgfs 1, 7, 8, 9, 10, 12 and 20 (5). Of these ligands, FgfrL1 appears to interact only with Fgf8 (16). One mechanism of action may therefore be that FgfrL1 binds to Fgf8 and serves as a co-receptor, presenting this ligand to Fgfr1. The activation of Fgfr1 would then lead to downstream signaling events that ultimately allow the survival of cells in the induced metanephric mesenchyme and the inhibition of apoptosis. In fact, the phenotypes of FgfrL1- and Fgf8-null mice are intriguingly similar (13,17,18). In both animal models, the development of nephrons is inhibited and increased apoptosis is observed in the metanephric mesenchyme. Another possibility may be that FgfrL1 is involved in the conversion of the induced metanephric mesenchymal cells into renal epithelial cells by controlling the alignment of mesenchymal cells. We have previously demonstrated that FgfrL1 can serve as an adhesion molecule if coated on bacterial plastic dishes (28). Moreover, it promotes cell-cell fusion if expressed at the surface of HEK293 cells and if mixed with CHO cells (29). It is possible that this cell fusion activity simply represents the ultimate stage of very tight cell-cell adhesion. FgfrL1 may therefore control the condensation of the metanephric mesenchyme around the ureteric tips by bringing together mesenchymal cells in an epithelial-like manner. The mechanisms by which Fgfr1 and Fgf8 are involved in this process are not yet fully elucidated. Further experiments are required to differentiate between all the possibilities outlined above.

## Figures and Tables

**Table I:** Primers used for preparation of hybridization probes.

Primer	Sequence	Accession no.
Calb1 up	ATGGATCCGACGGAAGTGGTTACCTGGA	
Calb1 low	TATCTAGATAAGAGCAAGGTCTGTTCGGTA	NM_009788
Fgfr1 up	GTGGATCCTCTTCTGGGCTGTGCT	
Fgfr1 low	ATTCTAGACCAGGGCCACTGTCTTGT	NM_010206
Fgfr2 up	GAGGATCCACCAACCAAATACCAAATC	
Fgfr2 low	TGTCTAGACCGTTCAACGACATCGAG	NM_010207
Fgfr3 up	GAGGATCCTGGTCCAGAGCAGCG	
Fgfr3 low	ACGTCTAGACCTAGAATGGCTGTCTGG	NM_008010
Fgfr4 up	GTGGATCCTGGATGACTCCTTAACCTC	
Fgfr4 low	TGTCTAGAGGGGTTGCTGTTGTCCAC	NM_008011

Underlined bases indicate restriction sites used for subcloning.

**Table II:** Phenotype of embryos and kidneys after the targeted inactivation of Fgfrs.

Receptor	Mouse phenotype (general KO)	Kidney phenotype (conditional KO for Fgfr1, Fgfr2)
Fgfr1	Lethal E7.5–E9.5	Normal
Fgfr2	Lethal E4–E10	Abnormalities in ureteric branching
Fgfr3	Abnormally long bones	Normal
Fgfr4	Normal	Normal
FgfrL1	Perinatally lethal (P0–P1)	Absence of nephrons

KO, knockout mice.

## Abbreviations

FGF: fibroblast growth factor (human)
Fgf: fibroblast growth factor (mouse)
FGFR: fibroblast growth factor receptor (human)
Fgfr: fibroblast growth factor receptor (mouse)
FGFRL1: fibroblast growth factor receptor-like protein 1
